# Strain-Induced Plasmon Confinement in Polycrystalline Graphene

**DOI:** 10.1021/acsphotonics.2c01157

**Published:** 2023-01-12

**Authors:** Simone Zanotto, Luca Bonatti, Maria F. Pantano, Vaidotas Mišeikis, Giorgio Speranza, Tommaso Giovannini, Camilla Coletti, Chiara Cappelli, Alessandro Tredicucci, Alessandra Toncelli

**Affiliations:** † NEST, Istituto Nanoscienze − CNR and Scuola Normale Superiore, Piazza S. Silvestro 12, Pisa, 56127, Italy; ‡ Scuola Normale Superiore, Piazza dei Cavalieri 7, Pisa, 56126, Italy; § Department of Civil, Environmental and Mechanical Engineering, University of Trento, Via Mesiano 77, Trento, 38123, Italy; ∥ Center for Nanotechnology Innovation @NEST - Istituto Italiano di Tecnologia, Piazza S. Silvestro 12, Pisa, 56127, Italy; ⊥ Centre for Materials and Microsystems, Fondazione Bruno Kessler, via Sommarive 18, Trento, I-38123, Italy; # Dipartimento di Fisica ”E. Fermi” and CISUP, Università di Pisa, and Istituto Nanoscienze - CNR, Largo Pontecorvo 3, Pisa, 56127, Italy

**Keywords:** graphene, terahertz, plasmons, strain, Drude-Smith, conductivity of polycrystalline 2D materials, atomistic simulations

## Abstract

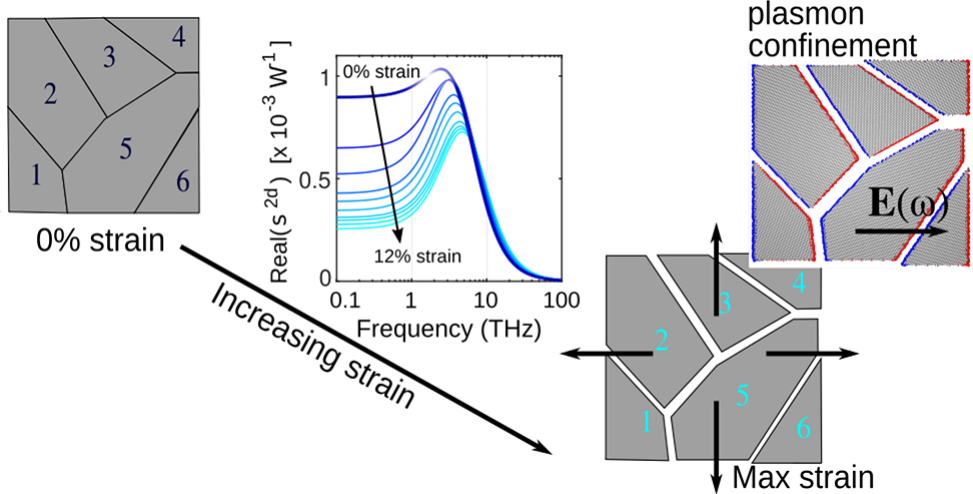

Terahertz spectroscopy is a perfect tool to investigate the electronic intraband conductivity of graphene, but a phenomenological model (Drude-Smith) is often needed to describe disorder. By studying the THz response of isotropically strained polycrystalline graphene and using a fully atomistic computational approach to fit the results, we demonstrate here the connection between the Drude-Smith parameters and the microscopic behavior. Importantly, we clearly show that the strain-induced changes in the conductivity originate mainly from the increased separation between the single-crystal grains, leading to enchanced localization of the plasmon excitations. Only at the lowest strain values explored, a behavior consistent with the deformation of the individual grains can instead be observed.

## Introduction

Extremely simple in its crystal structure, graphene has revealed an outstandingly complex and rich spectrum of fundamental physical phenomenologies, with unique perspectives toward technological applications.^1^ One of such peculiar features is the presence of an effective gauge field – analogous to the electromagnetic vector potential – when a strain is applied to an otherwise homogeneous, unperturbed graphene lattice.^2^ If an inhomogeneous strain belonging to a specific symmetry class is considered, the effective gauge field can be linked to a homogeneous pseudomagnetic field (PMF), capable – on par with a real magnetic field – of reorganizing the electronic states into discrete levels with a characteristic  energy behavior.^3,4^ The possibility of controlling electronic properties through strain, which gave birth to a whole field of research usually named straintronics,^5−9^ in graphene coexists with many other appealing properties of such material, like high carrier mobility, universal optical conductance, tunability by electrostatic and ionic gating, mechanical flexibility, and peculiar plasmonic properties. It is especially in the terahertz (THz) frequency range that graphene plasmonics and magnetoplasmonics deserve special attention, thanks to the small plasmon confinement length and high Faraday rotation power, enabling applications such as modulators, nonreciprocal elements, coherent absorbers, polaritonic components, detectors, and metasurfaces.^10−15^ Considering strained graphene, THz can be regarded both as an application domain and as a diagnostic tool. From the first perspective, THz manipulation can benefit from appropriate graphene strain engineering;^16^ in addition, pseudo-LL could be employed for ultrastrong coupling experiments, without the need for high magnetic field environments.^17−20^ From the second, one may leverage on the various THz spectroscopic techniques that proved powerful in understanding the micro- and mesoscopic structure of materials.^21,22^ Indeed, technologies to fabricate graphene samples of sufficient quality and size with an engineered strain are currently still challenging. On the other hand, the use of THz as a diagnostic tool has proven often extremely powerful. In the present article we investigate the THz conductivity of a variably strained polycrystalline graphene sample, unveiling the origin of the observed Drude-Smith behavior.^23−25^ We analyzed our experimental data by means of an atomistic technique, ωFQ,^26−28^ highlighting the presence of strain-induced carrier reflection at the grain boundaries and strain-induced mutual separation between grains. The ensuing localization of the plasmon excitations confirms that the conductivity trend of the macroscopic Drude-Smith model finds its roots in the creation of actual atom-size gaps between graphene grains of realistic size. Our study extends prior knowledge on the THz conductance of strained graphene where uniaxial strain was considered,^29,30^ but, most importantly, reports for the first time the application of a fully atomistic approach to quantify the deviation from the Drude model and, at the same time, provides a novel microscopic interpretation of the Drude-Smith behavior, which is a widespread tool for the analysis of THz conductance in nanostructured disordered materials.

## Results and Discussion

**Figure 1:**
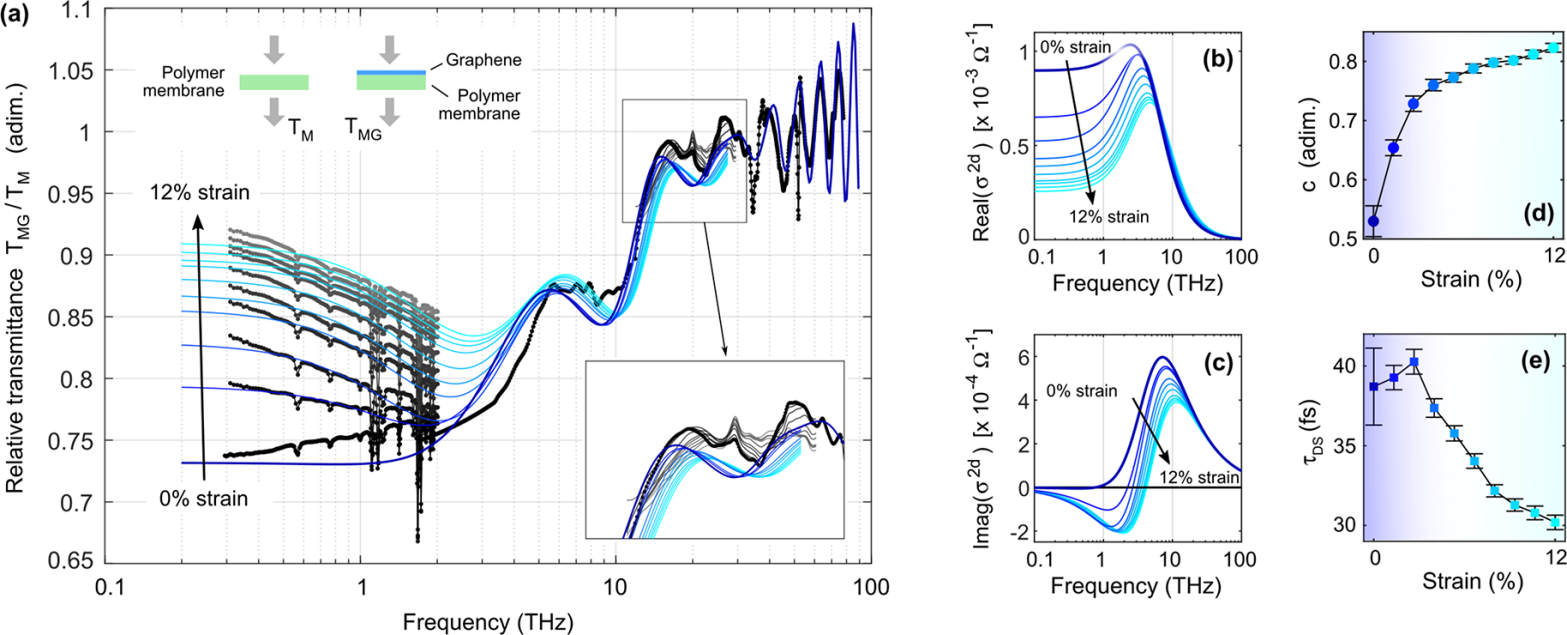
Spectroscopic evidence of the Drude-Smith behavior observed in the far-infrared response of strained polycrystalline graphene. (a) Experimental (black to gray dotted traces) and calculated (blue to cyan solid lines) relative transmittance spectra, defined as the ratio between the transmittance of the investigated sample (graphene on polymer membrane) and a reference sample (bare polymer membrane). The spectral region mostly affected by the Drude-Smith strain-dependent response is that between 0.2 and 3 THz. The inset shows a small effect mostly due to membrane thickness reduction. (b, c) Real and imaginary parts of graphene conductivity giving rise to the calculated spectra in (a). The suppression of Re(σ) at low frequencies is a fingerprint of Drude-Smith behavior. (d, e) Strain dependence of the Drude-Smith model parameters *c* (quantifying the carrier backscattering) and τ_DS_ (effective scattering time). Large strain imply stronger backscattering and smaller effective scattering time (more on this in the main text).

To perform the aforementioned study, we have designed a special sample and sample holder. Sample fabrication began with chemical vapor deposition (CVD) synthesis of a polycrystalline monolayer graphene film on copper foil (Alfa-Aesar, 25 μm thick) using an Aixtron BM Pro cold-wall CVD reactor. Wet transfer^31^ was used to deposit the graphene film on a thin (≈8 μm thick) circular polyvinyl chloride (PVC)-based membrane of about 20 mm diameter. The membrane was cut from a foil by means of a hollow punch in order to provide the desired geometry. The graphene sample was spin-coated with a protective layer of poly(methyl methacrylate) (PMMA), and the copper was etched away using iron chloride (FeCl_3_). The remaining PMMA/graphene film was rinsed in deionized water several times and was picked up from water using the PVC membrane. The sample was dried under ambient conditions. A reference sample consisting of the sole PVC membrane was also fabricated. The sample holder is a manual chuck with four jaws, on which the membrane sample can be accommodated and fixed by means of glue. By actuating the chuck, the four jaws move apart from one another, thus applying a biaxial deformation field to the sample, as illustrated in Figure S1 in the Supporting Information (SI). The usage of a four-jaw chuck allows to deliver a mostly homogeneous and isotropic strain distribution, avoiding position-dependent strain profiles that could give rise to specific PMF effects. In any case, it should be highlighted that the polycrystallinity of our sample prevents the creation of directionality-dependent effects, as the unstrained sample is isotropic. The sample is then inserted in the main optical path of two kinds of spectrometers: (i) a THz-TDS instrument, operating in the 0.3–2 THz spectral range, (ii) a FTIR instrument, operating either in the FIR (2–10 THz) or in the MIR (10–100 THz) spectral ranges depending on the employed beam splitter and detector. In Figure 1a we plot, as traces with black to gray color, the transmittance spectra collected under different strain levels, from 0 to 12%. For strained samples, only the 0.3–2 THz and 10–100 THz regions have been characterized, owing to setup limitations. The strain strongly affects the 0.3–2 THz region, while the 10–100 THz region is affected only weakly. All the spectra in Figure 1a are of relative transmittance, in the sense that they are the ratio between the spectra of a polymer+graphene sample with respect to the reference polymer sample. To interpret the spectra, we modeled the transmittance employing the scattering-matrix method for electromagnetic wave propagation through unpatterned layered media, following the theory in ref (32) after reduction to a single spatial harmonic, and generalization of the interface matrix to account for graphene as a zero-thickness sheet with local conductivity.^1^ As a first attempt, we employed a pure Drude form for the graphene conductivity; with this approach, however, it was not even possible to fit the zero-strain spectrum with reasonable parameters. The simple Drude form would be the (asymptotic) form expected for the conductivity of graphene in the frequency range below the onset of interband transitions, i.e., in the case of a sample grown with our technique, for frequencies smaller than around 150 THz.^33^ A deviation from the Drude behavior is however expected in polycrystalline samples; indeed, the Drude model is unable to capture the backscattering of carriers at the grain boundaries, as it only describes a time-domain Poissonian process of inelastic scattering events that is suitable for describing bulk scattering only. We thus moved to the Drude-Smith model, that, for graphene, is given by the expression
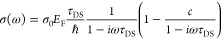
1where σ_0_ = 2*e*^2^/*h* ≈ 7.75 × 10^–5^ Ω^–1^ is the conductance quantum, *E*_F_ is the Fermi energy, τ_DS_ is the Drude-Smith scattering time (related, but distinct from the bulk scattering time), and *c* ∈ [0, 1] is a parameter that quantifies the deviation from the pure Drude model.^23^ The choice *c* = 0 recovers the Drude model; the effect of *c* > 0 is to suppress the DC conductivity and to move the maximum of Re(σ) from ω = 0 (as in the Drude model) to a finite value ω > 0. Such effects are interpreted, in the original view of the model given by Smith, as a “memory effect” experienced by the carriers, whose velocity distribution is not fully randomized after the first collision, and rather retains a net backward component.^23^ Otherwise, it can be interpreted as the consequence of a finite reflectivity felt by the carriers while moving across the sample, modeled as a disordered assembly of grains with partially impenetrable boundaries.^24^

The transmittance spectra following from the fit to experimental data using our multilayer model and eq 1 for the graphene conductivity are reported in Figure 1a as blue to cyan lines. No data are shown for the strained samples above 30 THz since in that spectral region one only observes a shift of the oscillations, an effect connected to the change of the polymer thickness rather than to a modification of the Drude-Smith parameters of graphene. More on this, and additional information on the fitting procedure, are given in SI, section 2. The slight increase with frequency in the 0.5–2 THz range (of the order of just a few percent) in the experimental transmission for the unstrained spectrum is likely due to deviations from flatness of the membrane in the absence of any applied tension (with possible formation of small bends and wrinkles) or to residuals of the polymer absorption. Consistently with the general behavior of the Drude-Smith model with nonzero *c*, the real part of the conductance has a maximum at finite values of the frequency (≈3 THz, see Figure 1b). As the strain increases, the DC conductance decreases, reducing absorption at low frequency. This is a consequence of *c* shifting from 0.5 to 0.82, which means a strong deviation from the Drude model at large strains (Figure 1d). A peculiar trend is also observed in the τ_DS_ parameter (Figure 1e). Here, the best estimates for τ_DS_ first increase and then decrease; however, especially when considering the data with error bars (95% CI statistical error), the initial increasing τ_DS_ trend is not unambiguous and the data could also be consistent with a global decrease, albeit with different slopes for strains below or above ≈3.5%. A microscopic interpretation of this effect is given later. The data of Figure 1 complement literature reports about the conductance in strained graphene, where either the maximum strain was much smaller than ours^30^ or uniaxial strain was employed.^29^

To move toward a microscopic interpretation of the trends observed in Figure 1d,e, one can resort to the model proposed by Cocker et al.,^24^ where the carriers motion in a multidomain sample is studied by a Montecarlo approach and by considerations on the diffusion current. In this model, new parameters are involved: the diffusion time in each domain (*t*_0_), the domain wall reflectivity (*R*), and the bulk scattering time (τ). However, the two models are connected, since relatively simple algebraic relations between the above-mentioned parameters and the “original” Drude-Smith parameters (*c*, τ_DS_) can be derived. Exploiting such relations, we report in the SI (section 3.6) details on the analysis of our data in view of the model by Cocker et al. In summary, the domain diffusion time decreases with increasing strain, the reflectivity increases with increasing strain, and the bulk scattering time remains almost constant. These observations support the vision that the strain applied to the polycrystalline sample leads to a progressive separation between the monocrystals (connected to reflectivity increase) and to the creations of cracks within each crystal (connected to diffusion time decrease, assuming that diffusion time equals a drift velocity multiplied by the domain size). Effects connected to the stretch of carbon–carbon bonds seem instead negligible^34^ (see section 3.8 in the SI), and the consequent opening of a bandgap, yet expected at even larger strains, would rather affect mostly the optical spectral region.^35^ It should be recalled, however, that the model by Cocker et al. was derived for massive charges, which is not the case of graphene.

**Figure 2:**
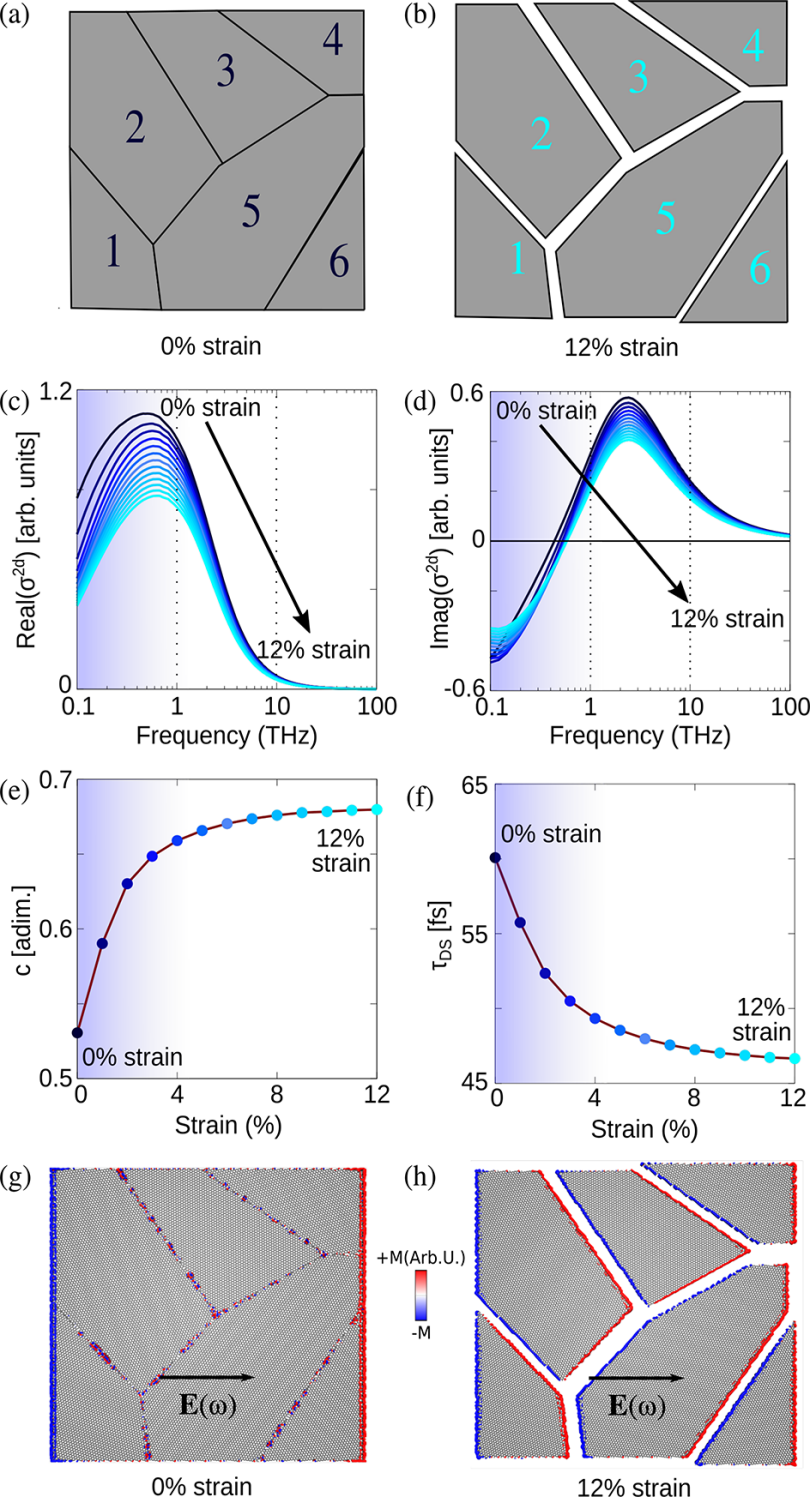
Polycrystalline graphene sheet composed of 6 grains at rest (a) and under the effect of biaxial strain (b). ωFQ values for the real (c) and imaginary (d) part of σ^2*d*^ as a function of the external frequency. Drude-Smith parameters *c* (e) and τ_DS_ (f) as a function of the applied strain. Charge density plots for the system at rest (g) and at 10% strain (h).

To provide a more solid analysis of Dirac Fermions dynamics in a multidomain graphene sample, we then exploit a fully atomistic, yet classical, model, called ωFQ,^26−28,36^ which is able to correctly describe how the grain boundaries morphology affects the electron conduction across the sample (see also Methods).^37^ In ωFQ, each carbon atom is endowed with a net charge *q*_*i*_, which responds to an external oscillating electric field. The conduction properties of graphene samples are directly obtained from computed ωFQ charges, by exploiting their complex (i.e., real + imaginary) nature. In fact, the charge’s imaginary part enters the definition of the dipole moment of the whole system and, as a consequence, the calculation of the complex polarizability (α(ω)), that can be easily related to the conductivity (see section 3.1 in the SI). Also, the charge exchange between nearest neighbor atoms is described in terms of the Drude model of conductance, which is further modulated by means of a Fermi-like damping function, which introduces an exponential decay, typical of quantum tunneling (see Methods section).^26,27^ In this work, ωFQ needs to be applied to graphene samples with a size of tens of μm. Although ωFQ can afford graphene sheets constituted by more than 1 million atoms due to its low computational cost,^28^ a fully atomistic modeling of graphene samples of size of the order of μm is still computationally unfeasible. However, it has been shown that by modulating the graphene 2D electron density, or equivalently its Fermi level, the plasmonic properties of a graphene sheet of arbitrary size can be reproduced.^38^ Indeed, ωFQ is able to correctly reproduce the peculiar property of graphene-based materials to yield plasmon degeneracy (i.e., the same Plasmon Resonance Frequency - PRF) by modulating both the intrinsic dimensions of the considered substrate and the Fermi energy by the same numerical factor.^27,38^ In this scenario, a graphene-based nanostructure showing a PRF in the THz regime, which is unfeasible from a computational point of view due to the prohibitive number of atoms to be described, can be modeled as a smaller system by decreasing the Fermi level to make the electron density equal to that of the real-size structure. Remarkably, ωFQ retains this important physical feature;^27^ therefore, in the present work, we adopt a special parametrization, which is able to correctly describe the initial experimental system at rest (see section 3.2 in the SI). In this way, graphene samples characterized by a PRF in the THz regime can be described by actually computing much smaller structures (i.e., tens of nm) and the results of calculations can be directly compared with experimental data. We model the experimental unstressed polycrystalline graphene as a multidomain graphene sheet constituted of 6 grains of different size as the starting geometry at rest (see Figure 2 a). Obviously, multiple starting structures could be exploited; additional results obtained by using a multidomain graphene sheet constituted of 12 and 17 grains are reported in section S3.7 in the SI. Such data show that similar trends are obtained independently of the number of grains. Drude-Smith parameters are extrapolated from conduction trends with a fitting procedure (see section 3.3 in the SI). Also, in order to allow for a direct comparison between our modeling and the experimental data, we impose the Drude-smith parameter *c* = 0.53 at 0% strain (see eq 1 and Figure 1 d), by tuning the free parameter β (see sections 3.2 and 3.4 in the SI). ωFQ is then tasked to explain at the microscopic level experimental macroscopic trends as a function of the applied strain (see Figure 1). We first assume that, by starting from the unstressed polycrystalline graphene, in which all grains are linked together, the grains start to drift away as the applied strain increases. Thus, gaps between adjacent grains are first formed and then their size increases as a function of the applied strain, so that cracks between grains appear (see Figure 2a,b). Computed ωFQ real and imaginary parts of the conductivity (σ^2*d*^) for each strained geometry (from 0% to 12% external applied strain, with a constant step of 1%) are reported in Figure 2c and d, respectively.

The ωFQ Re(σ^2*d*^) maximum for graphene at rest falls at about 0.7 THz, which differs from the experimental result. However, such a shift does not affect the overall description of the behavior of the conduction peak as a function of the applied strain. In fact, as reported in Figure 1c,d, the conductivity blue shifts as the applied strain increases, coherently with experimental findings. In addition, ωFQ reproduces well the experimental decrease of the peak’s intensity as the applied strain increases.

By exploiting the fitting procedure explained in section 3.3 in the SI, the *c* and τ_DS_ parameters entering the Drude-Smith model (see eq 1) can be obtained as a function of the applied strain (see Figure 2e,f). Also in this case, the agreement between ωFQ results and the experiment is extremely good. Indeed, not only the general behavior as a function of the strain is correctly reproduced for applied strains >2–3%, but also ωFQ results are numerically comparable with experiments. These findings are particularly relevant, because they confirm the reliability of our modeling of the experimental strain process. The decreasing of τ_DS_ as the applied strain increases can be explained by considering that for large strains, grains tend to behave as independent islands and the electron conduction between adjacent domains is strongly damped. As a result, the plasmonic density is blocked inside each single grain and the overall scattering time drops. In Figure 2g,h, we show that the charge density is diffused over the whole unstressed sample, whereas a confinement of the plasmon density arises when gaps originate in the structure. Although we are able to mimic with very good accuracy the experimental trends for strains >3.5%, our theoretical picture does not capture the change in the slope of τ_DS_ as a function of strain occurring when we move to values below ∼3.5%. Such a discrepancy probably reflects that in weakly stressed graphene an additional relaxation mechanism, more relevant than grain separation, is playing an active role. In fact, the results reported in Figure 2c–f are obtained by assuming the internal relaxation of the C–C bonds within each grain to be instantaneous. This is an approximation which may be valid for large strain values, but may fail at low strain values (1–3%).

**Figure 3:**
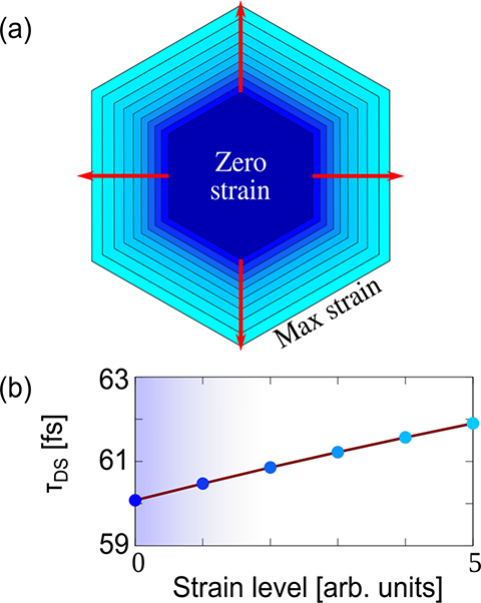
(a) Graphical representation of the elongation of a single graphene ring under the effect of an increasing level of isotropic strain. (b) Drude-Smith parameter τ_DS_ as a function of the applied strain by imposing a stretching of C–C bonds only.

To support this vision, we consider the alternative situation produced by the strain: the multidomain unstressed graphene is elongated, stretching the chemical bonds without detaching the grains (see Figure 3a and section 3.8 in the SI). At the grain boundaries amorphous C phases could indeed be present, and they might prevent a clear separation of the islands until the strain is large enough, leading to a complex interplay of mechanisms. If grains deform without moving away from each other, an overall increase of the system size, and, as a consequence, of the scattering time, is expected, without major changes in the plasmon confinement. In fact, the computed ωFQ trend of τ_DS_ as a function of the applied strain shows in this case a small linear increase (see Figure 3b). Such findings support the experimental observations that, for strains lower than 3.5%, the values of τ_DS_ are relatively unchanged (within the errors).

In summary, two different mechanisms have been investigated by our computational modeling: the elongation of the C–C bonds in the whole polycrystalline structure and the formation of different cracks across the grain boundaries of the structure, leading to narrow gaps between adjacent grains, providing a confinement of the plasmons within the different grains. Opposite τ_DS_ trends are obtained from the simulations in the two cases, which allows interpreting the experimental results as deriving from the interplay of the two processes: while at low applied strains both elongation of the C–C bonds’ length and separation between adjacent grains may be involved, at high levels of strain, the latter effect clearly dominates the THz response.

## Conclusions

We have investigated the THz conductivity of polycrystalline graphene as a function of isotropically applied strain by both time domain/FTIR spectroscopy and theoretical atomistic simulations. It is found that the dependence can be properly described by considering two different effects: a uniform deformation of the graphene lattice, which dominates for low strain, and a progressive detachment of the individual monocrystal grains at high strain. The latter affects the THz conductivity through the progressive localization of plasmon excitations in each grain.

Furthermore, by fitting both experimental and theoretical spectra with a phenomenological Drude-Smith model, a connection is drawn between the parameters accounting for disorder in the formula and the microscopic physics they are expected to convey.

The results highlight the potential of the ωFQ approach in studying systems where macroscopic electro-dynamic theories cannot cope with the presence of underlying micro/nanoscale structures at the atomic scale, if not through the introduction of average parameters of limited quantitative significance. They also finally provide a clear picture of how polycrystalline disorder affects graphene plasmon resonances, suggesting a new interesting mechanism by which strain could be used as the external control knob in the implementation of graphene-based tunable THz devices.

## Methods

In this work, we describe the optical properties of multidomain graphene nanostructures, with an emphasis on structural defects created by a biaxial strain applied to a sample initially at rest. We model the optical response of such structures by using a classical, fully atomistic approach, called ωFQ.^26−28,36,37,39^ In this method, a net complex charge *q*_*i*_ is placed at each atomic site, and atom–atom charge flow occurs in response to a time dependent external electric field. Charge exchange is described in terms of the Drude model of conduction, modulated by quantum tunneling effects. In this way, charge transfer is restricted to nearest neighboring atoms, and the typical quantum tunneling exponential decay is considered. ωFQ charges (**q**) are determined through the following equation:^27^
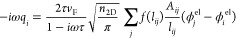
2where *q*_*i*_(ω) is a complex-valued charge placed at atom *i*, oscillating at frequency ω. *v*_F_ is the Fermi velocity, *n*_2D_ is the 2D-density of graphene, and τ the scattering time. *l*_*ij*_ is the distance between atoms *i* and *j*, whereas *A*_*ij*_ is the effective area connecting *i* and *j* atoms. The electrochemical potential acting on each atomic site is labeled as ϕ^el^. Finally, *f*(*l*_*ij*_) is a Fermi-like damping function mimicking quantum tunneling effects, which reads as the following:
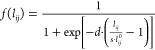
3where *l*_*ij*_^0^ is the equilibrium distance between two adjacent carbon atoms at rest (i.e., *l*_*ij*_^0^ = 1.42 Å^40^). The position of the inflection point and the steepness of the step function are determined by the parameters *d* and *s*, respectively.

## Supporting Information Available

Details on the sample holder used during the experimental procedure and on the fitting procedure for the experimental data. ωFQ equations for conductivity and parametrization of the system. Computational details. Comparison between experimental and computational trends for Cocker parameters. Role of the grain size on the Drude-Smith and Cocker parameters. Alternative strain modeling: elongation of C–C bonds (PDF)

## References

[ref1] Ferrari A. C.; Bonaccorso F.; Fal’Ko V.; Novoselov K. S.; Roche S.; Bøggild P.; Borini S.; Koppens F. H.; Palermo V.; Pugno N.; et al. Science and technology roadmap for graphene, related two-dimensional crystals, and hybrid systems. Nanoscale 2015, 7, 4598–4810. 10.1039/C4NR01600A. 25707682

[ref2] Guinea F.; Katsnelson M.; Geim A. Energy gaps and a zero-field quantum Hall effect in graphene by strain engineering. Nat. Phys. 2010, 6, 30–33. 10.1038/nphys1420.

[ref3] Levy N.; Burke S.; Meaker K.; Panlasigui M.; Zettl A.; Guinea F.; Neto A. C.; Crommie M. F. Strain-induced pseudo–magnetic fields greater than 300 T in graphene nanobubbles. Science 2010, 329, 544–547. 10.1126/science.1191700. 20671183

[ref4] Nigge P.; Qu A.; Lantagne-Hurtubise É.; Mårsell E.; Link S.; Tom G.; Zonno M.; Michiardi M.; Schneider M.; Zhdanovich S.; et al. Room temperature strain-induced Landau levels in graphene on a wafer-scale platform. Science Advances 2019, 5, eaaw5593 10.1126/sciadv.aaw5593. 31723598 PMC6839937

[ref5] Bukharaev A. A.; Zvezdin A. K.; Pyatakov A. P.; Fetisov Y. K. Straintronics: a new trend in micro-and nanoelectronics and materials science. Physics-Uspekhi 2018, 61, 1175 10.3367/UFNe.2018.01.038279.

[ref6] Si C.; Sun Z.; Liu F. Strain engineering of graphene: a review. Nanoscale 2016, 8, 3207–3217. 10.1039/C5NR07755A. 26796960

[ref7] Zhu S.; Stroscio J. A.; Li T. Programmable extreme pseudomagnetic fields in graphene by a uniaxial stretch. Physical review letters 2015, 115, 245501 10.1103/PhysRevLett.115.245501. 26705640 PMC4711939

[ref8] Zhang D.-B.; Seifert G.; Chang K. Strain-induced pseudomagnetic fields in twisted graphene nanoribbons. Physical review letters 2014, 112, 096805 10.1103/PhysRevLett.112.096805. 24655271

[ref9] Castro-Villarreal P.; Ruiz-Sánchez R. Pseudomagnetic field in curved graphene. Phys. Rev. B 2017, 95, 125432 10.1103/PhysRevB.95.125432.

[ref10] Li Y.; Tantiwanichapan K.; Swan A. K.; Paiella R. Graphene plasmonic devices for terahertz optoelectronics. Nanophotonics 2020, 9, 1901–1920. 10.1515/nanoph-2020-0211.

[ref11] Liu P. Q.; Luxmoore I. J.; Mikhailov S. A.; Savostianova N. A.; Valmorra F.; Faist J.; Nash G. R. Highly tunable hybrid metamaterials employing split-ring resonators strongly coupled to graphene surface plasmons. Nat. Commun. 2015, 6, 1–7. 10.1038/ncomms9969. PMC4673875 26584781

[ref12] Tamagnone M.; Moldovan C.; Poumirol J.-M.; Kuzmenko A. B.; Ionescu A. M.; Mosig J. R.; Perruisseau-Carrier J. Near optimal graphene terahertz non-reciprocal isolator. Nat. Commun. 2016, 7, 1–6. 10.1038/ncomms11216. PMC4823866 27048760

[ref13] Asgari M.; Riccardi E.; Balci O.; De Fazio D.; Shinde S. M.; Zhang J.; Mignuzzi S.; Koppens F. H.; Ferrari A. C.; Viti L.; et al. Chip-Scalable, Room-Temperature, Zero-Bias, Graphene-Based Terahertz Detectors with Nanosecond Response Time. ACS Nano 2021, 15, 17966–17976. 10.1021/acsnano.1c06432. 34706194 PMC8613901

[ref14] Zanotto S.; Bianco F.; Miseikis V.; Convertino D.; Coletti C.; Tredicucci A. Coherent absorption of light by graphene and other optically conducting surfaces in realistic on-substrate configurations. APL Photonics 2017, 2, 016101 10.1063/1.4967802.

[ref15] Miao Z.; Wu Q.; Li X.; He Q.; Ding K.; An Z.; Zhang Y.; Zhou L. Widely tunable terahertz phase modulation with gate-controlled graphene metasurfaces. Physical Review X 2015, 5, 041027 10.1103/PhysRevX.5.041027.

[ref16] Nguyen V. H.; Lherbier A.; Charlier J.-C. Optical Hall effect in strained graphene. 2D Materials 2017, 4, 025041 10.1088/2053-1583/aa5f8b.

[ref17] Hagenmüller D.; Ciuti C. Cavity QED of the graphene cyclotron transition. Phys. Rev. Lett. 2012, 109, 267403 10.1103/PhysRevLett.109.267403. 23368618

[ref18] Chirolli L.; Polini M.; Giovannetti V.; MacDonald A. H. Drude weight, cyclotron resonance, and the Dicke model of graphene cavity QED. Physical review letters 2012, 109, 267404 10.1103/PhysRevLett.109.267404. 23368619

[ref19] Pellegrino F.; Chirolli L.; Fazio R.; Giovannetti V.; Polini M. Theory of integer quantum Hall polaritons in graphene. Phys. Rev. B 2014, 89, 165406 10.1103/PhysRevB.89.165406.

[ref20] Mann C.-R.; Horsley S. A.; Mariani E. Tunable pseudo-magnetic fields for polaritons in strained metasurfaces. Nat. Photonics 2020, 14, 669–674. 10.1038/s41566-020-0688-8.

[ref21] Ulbricht R.; Hendry E.; Shan J.; Heinz T. F.; Bonn M. Carrier dynamics in semiconductors studied with time-resolved terahertz spectroscopy. Rev. Mod. Phys. 2011, 83, 543 10.1103/RevModPhys.83.543.

[ref22] Buron J. D.; Petersen D. H.; Bøggild P.; Cooke D. G.; Hilke M.; Sun J.; Whiteway E.; Nielsen P. F.; Hansen O.; Yurgens A.; et al. Graphene conductance uniformity mapping. Nano Lett. 2012, 12, 5074–5081. 10.1021/nl301551a. 22947167

[ref23] Smith N. Classical generalization of the Drude formula for the optical conductivity. Phys. Rev. B 2001, 64, 155106 10.1103/PhysRevB.64.155106.

[ref24] Cocker T. L.; Baillie D.; Buruma M.; Titova L. V.; Sydora R. D.; Marsiglio F.; Hegmann F. A. Microscopic origin of the Drude-Smith model. Phys. Rev. B 2017, 96, 205439 10.1103/PhysRevB.96.205439.

[ref25] Joyce H. J.; Boland J. L.; Davies C. L.; Baig S. A.; Johnston M. B. A review of the electrical properties of semiconductor nanowires: insights gained from terahertz conductivity spectroscopy. Semicond. Sci. Technol. 2016, 31, 103003 10.1088/0268-1242/31/10/103003.

[ref26] Giovannini T.; Rosa M.; Corni S.; Cappelli C. A classical picture of subnanometer junctions: an atomistic Drude approach to nanoplasmonics. Nanoscale 2019, 11, 6004–6015. 10.1039/C8NR09134J. 30869089

[ref27] Giovannini T.; Bonatti L.; Polini M.; Cappelli C. Graphene Plasmonics: Fully Atomistic Approach for Realistic Structures. journal of physical chemistry letters 2020, 11, 7595–7602. 10.1021/acs.jpclett.0c02051. 32805117 PMC7503861

[ref28] Lafiosca P.; Giovannini T.; Benzi M.; Cappelli C. Going beyond the limits of classical atomistic modeling of plasmonic nanostructures. J. Phys. Chem. C 2021, 125, 23848–23863. 10.1021/acs.jpcc.1c04716. PMC8573767 34765073

[ref29] Kim J.; Lee C.; Bae S.; Jin Kim S.; Soo Kim K.; Hee Hong B.; Choi E. Effect of uni-axial strain on THz/far-infrared response of graphene. Appl. Phys. Lett. 2012, 100, 041910 10.1063/1.3680095.

[ref30] Chhikara M.; Gaponenko I.; Paruch P.; Kuzmenko A. B. Effect of uniaxial strain on the optical Drude scattering in graphene. 2D Materials 2017, 4, 025081 10.1088/2053-1583/aa6c10.

[ref31] Li X.; Cai W.; An J.; Kim S.; Nah J.; Yang D.; Piner R.; Velamakanni A.; Jung I.; Tutuc E.; et al. Large-area synthesis of high-quality and uniform graphene films on copper foils. science 2009, 324, 1312–1314. 10.1126/science.1171245. 19423775

[ref32] Whittaker D.; Culshaw I. Scattering-matrix treatment of patterned multilayer photonic structures. Phys. Rev. B 1999, 60, 2610 10.1103/PhysRevB.60.2610.

[ref33] Ivanov I.; Bonn M.; Mics Z.; Turchinovich D. Perspective on terahertz spectroscopy of graphene. EPL (Europhysics Letters) 2015, 111, 67001 10.1209/0295-5075/111/67001.

[ref34] Pellegrino F.; Angilella G.; Pucci R. Strain effect on the optical conductivity of graphene. Phys. Rev. B 2010, 81, 035411 10.1103/PhysRevB.81.035411.

[ref35] Pereira V. M.; Castro Neto A. H.; Peres N. M. R. Tight-binding approach to uniaxial strain in graphene. Phys. Rev. B 2009, 80, 045401 10.1103/PhysRevB.80.045401.

[ref36] Bonatti L.; Gil G.; Giovannini T.; Corni S.; Cappelli C. Plasmonic Resonances of Metal Nanoparticles: Atomistic vs. Continuum Approaches. Frontiers in Chemistry 2020, 8, 340 10.3389/fchem.2020.00340. 32457870 PMC7221199

[ref37] Bonatti L.; Nicoli L.; Giovannini T.; Cappelli C. In silico design of graphene plasmonic hot-spots. Nanoscale Advances 2022, 4, 2294–2302. 10.1039/D2NA00088A. 35706845 PMC9113057

[ref38] Yu R.; Cox J. D.; Saavedra J.; Garcia de Abajo F. J. Analytical modeling of graphene plasmons. ACS Photonics 2017, 4, 3106–3114. 10.1021/acsphotonics.7b00740.

[ref39] Giovannini T.; Bonatti L.; Lafiosca P.; Nicoli L.; Castagnola M.; Illobre P. G.; Corni S.; Cappelli C. Do We Really Need Quantum Mechanics to Describe Plasmonic Properties of Metal Nanostructures?. ACS photonics 2022, 9, 3025–3034. 10.1021/acsphotonics.2c00761. 36164484 PMC9502030

[ref40] Castro Neto A. H.; Guinea F.; Peres N. M. R.; Novoselov K. S.; Geim A. K. The electronic properties of graphene. Reviews of Modern Physics 2009, 81, 109 10.1103/RevModPhys.81.109.

