# Supplementary material for: Strain-Induced Plasmon Confinement in Polycrystalline Graphene
Source: ACS Photonics. 2023 Jan 12;10(2):394–400. doi: 10.1021/acsphotonics.2c01157 (PMC9936574; doi:10.1021/acsphotonics.2c01157)
Supplement: Supplementary file 1 — ph2c01157_si_001.pdf [file ph2c01157_si_001.pdf]

Supporting Information: Strain-induced plasmon confinement in polycrystalline graphene

Simone Zanotto,[†] Luca Bonatti,[‡] Maria F. Pantano,[¶] Vaidotas Mišeikis,[§] Giorgio Speranza,[||] Tommaso Giovannini,[‡] Camilla Coletti,[§] Chiara Cappelli,[‡] Alessandro Tredicucci,[*,⊥] and Alessandra Toncelli[⊥]

†*NEST, Istituto Nanoscienze – CNR and Scuola Normale Superiore, Piazza S. Silvestro 12, Pisa, 56127, Italy.*

‡*Scuola Normale Superiore, Piazza dei Cavalieri 7, 56126 Pisa, Italy.*

¶*Department of Civil Environmental and Mechanical Engineering, University of Trento, Via Mesiano 77, Trento, 38123, Italy.*

§*Center for Nanotechnology Innovation @NEST - Istituto Italiano di Tecnologia, Piazza S. Silvestro 12, Pisa, 56127, Italy.*

||*Centre for Materials and Microsystems, Fondazione Bruno Kessler, via Sommarive 18, Trento, I-38123, Italy*

⊥*Dipartimento di Fisica "E. Fermi" and CISUP, Università di Pisa, and Istituto Nanoscienze - CNR, Largo Pontecorvo 3, 56127 Pisa, Italy.*

E-mail: alessandro.tredicucci@unipi.it

S1 Sample holder

In order to apply a strain to the sample that is as isotropic as possible, the membrane has been mounted on the four-jaw chuck represented in Fig. S1. Appropriate calibration allowed connecting the opening of the jaws, which are manually actuated, with the strain delivered to the membrane, in the region where it supports the graphene sample.

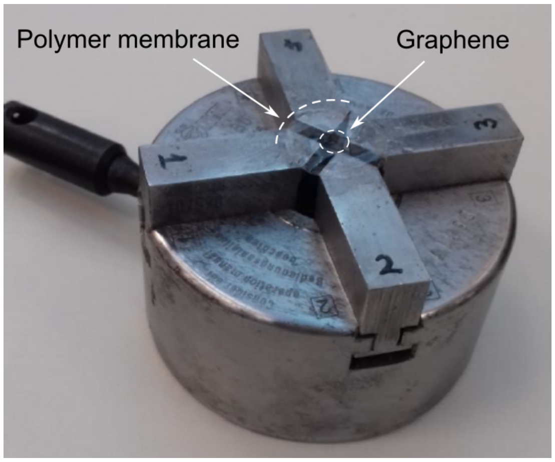

Figure S1: Picture of the manual chuck employed to apply strain on the graphene/polymer membrane sample.

S2 Details on the fitting procedure

The strain-dependent experimental data of Fig. 1(a) in the main text have been fitted by an electromagnetic scattering-matrix model for layered structures, where multiple parameters enter either directly (i.e. polymer membrane thickness d_{membr}) or indirectly (i.e. Drude-Smith parameters E_{F}, τ_{DS} and c, that enter through the conductivity as per Eq. 1 of the main text). While in principle the ideal procedure would have been to fit all the spectra leaving free all the above listed parameters, in practice it turned out that this procedure was not numerically stable. We hence reduced the fit complexity by first fitting only the zero strain spectrum using as free parameters the full set $\{d_{\text{membr}}, E_{\text{F}}, \tau_{\text{DS}}, \text{c}\}$. The best fit is obtained for $d_{\text{membr}} = 7.74\ \mu\text{m}, E_{\text{F}} = 406$ meV, $\tau_{\text{DS}} = 38.7$ fs, $c = 0.53$. Subsequently, we fitted the strain-dependent spectra over the sole mid-infrared ($10-100$ THz) spectral region using only d_{membr} as free parameter. Our ansatz was that, under our conditions, this spectral region is very weakly affected by the properties of graphene, as we eventually verified with an *a posteriori* check, see below. Indeed, the shift of Fabry-Pérot

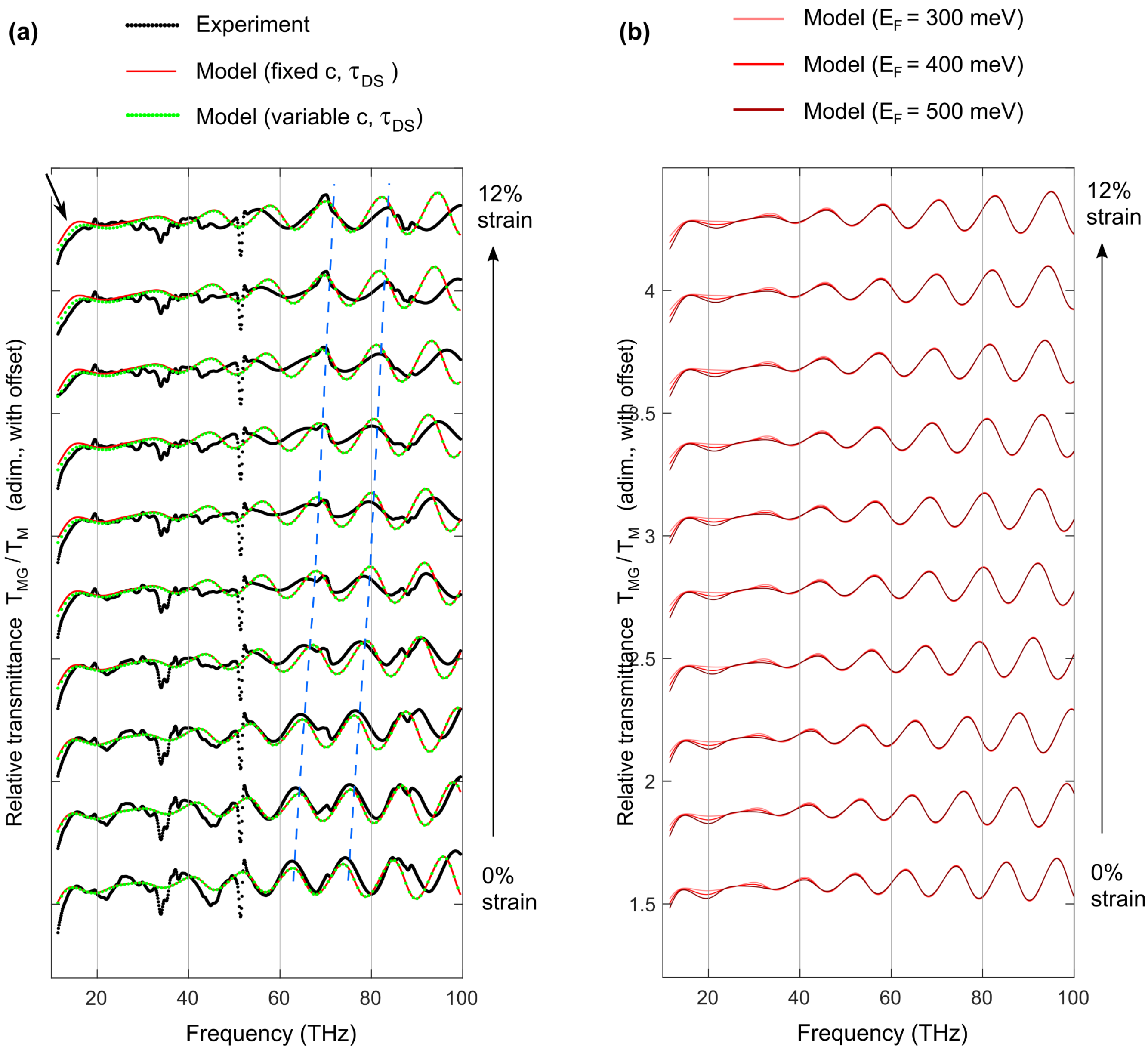

Figure S2: Fitting strain-dependent spectra over the mid-infrared spectral region.

oscillations observed in Fig. S2 (a) originates almost solely from a thickness reduction of the polymer. By this procedure we retrieved the following values for the polymer thickness, in correspondence to the $0\% \rightarrow 12\%$ strain sweep: $d_{\mathrm{membr}} = [7.74, 7.58, 7.50, 7.25, 7.17, 7.15, 7.10, 7.05, 7.00, 6.95]$ μm. This fit was performed manually, keeping fixed τ_{DS}, c and E_{F} at the zero-strain values, and varying d_{membr} in order to match the trend of the peaks highlighted by the blue dashed lines in Fig. S2 (a). We were limited to this quite narrow spectral range because of noise in the range $20-60$ THz (due to atmospheric absorption) and because

of additional fringe shift effects above 90 THz (due to the polymer refractive index dispersion). Using the now determined d_{membr} strain-dependent values, and fixing E_{F} to the zero-strain value ($E_{\text{F}} = 406$ meV), we finally fitted the strain-dependent data in the $0.3-2$ THz range using only τ_{DS} and c as parameters (results in main Fig. 1(d,e)). We eventually checked that variations of τ_{DS}, c and E_{F}, in their respective relevant ranges, have a negligible effect to the spectra in the $10-100$ THz region. In Fig. S2 (a) we report, superimposed to the experimental data, the calculated spectra, using the d_{membr} values listed above and either $\tau_{\text{DS}} = 38.7$ fs, $c = 0.53$ (i.e., values from the fit of zero-strain full-spectrum data) or the strain-dependent τ_{DS} and c (i.e., data in main Fig. 1(d,e)). It is evident that both approaches lead to almost identical curves, with a tiny deviation visible only in the 12% strain curve at low frequencies (see the difference between red curve and green points, highlighted by an arrow in top left of Fig. S2 (a)). Similarly, we analyzed the role of E_{F} (Fig. S2 (b)); here, a small Fermi-energy-related spectral change is visible in the $10-60$ THz range, but this range was not considered for the strain-dependent retrieval of d_{membr}. In summary, we justified our ansatz stated above, obtaining support to the solidity of the data reported in main Fig. 1.

S3 Methods: the ωFQ model

S3.1 Conductivity in ωFQ

ωFQ charges are obtained by solving Eq. 2 in the main text. Once the complex charges are known, the real and complex part of polarizability are easily recovered.[1]

The real and imaginary part of the conductivity at a certain frequency (ω) can be computed by assuming a non-local approximation (i.e. the local electric field at a certain atomic position is equal to the external applied electric field).

Within this approximation, and by considering a linear response regime, the induced polarization density **P** and the external electric field (**E**) are related by:

$$\mathbf{P} = \frac{\chi_e}{4\pi} \cdot \mathbf{E} \tag{S1}$$

where χ_e is the electric susceptibility. From a microscopic point of view, the relation between the dipole moment ($\mathbf{p}$) and the local electric field ($\mathbf{E}_{loc}$) reads:

$$\mathbf{p} = \frac{N \cdot \alpha \cdot \mathbf{E}_{loc}}{4\pi} \approx \frac{N \cdot \alpha \cdot \mathbf{E}}{4\pi} \tag{S2}$$

where N is the number density (i.e. number of atoms per unit surface). Thus, the relation between $\alpha(\omega)$ and the complex conductivity $\sigma(\omega)$ is:

$$\alpha(\omega) = \frac{\chi}{N} = \frac{\varepsilon(\omega) - 1}{N} = \frac{1 + \frac{4\pi i \sigma(\omega)}{\omega} - 1}{N} = \frac{4\pi i \sigma(\omega)}{\omega N} \tag{S3}$$

From Eq. S3, the real (σ_{Re}) and imaginary (σ_{Im}) parts of the conductivity can be obtained:

$$\sigma_{Re}(\omega) = \frac{N\omega\alpha_{Im}(\omega)}{4\pi} \qquad \sigma_{Im}(\omega) = -\frac{N\omega\alpha_{Re}(\omega)}{4\pi} \tag{S4}$$

S3.2 ωFQ in the THz regime

In the ωFQ model, the 2D electron density of graphene enters the definition of the equation of motion of the charges (see Eq. 2 in the main text). In particular, the 2D electron density n_{2D} for a graphene sheet is defined as the ratio between the number of atoms N_{atoms} and the surface of the considered sheet S:

$$n_{2D} = \frac{\beta \cdot N_{atoms}}{S} \tag{S5}$$

where β is a dimensionless factor representing the fraction of π electrons which are involved in the plasmon excitation.[1] The 2D electron density, and so the β parameter, is determined by the Fermi level E_F of the system by the following relation (in atomic units):[1]

$$E_F = v_F \sqrt{\pi n_{2D}} \tag{S6}$$

where v_F is the Fermi velocity (10^6 m/s). The ability of ωFQ to tune the PRF as a function of E_F (i.e. by adjusting the β parameter) is reported in Fig. S3-a, in which the PRF of a graphene disk with a

diameter D equal to 25 nm, is studied for several values of E_F (from E_F = 0.2 to E_F = 1.0 eV, with a step of 0.2 eV). In particular, the PRF blue-shifts by increasing E_F, reproducing a trend already reported in literature from graphene nanostructures.[1–5] In addition, the PRF of graphene disks can be red/blue-shifted by increasing/decreasing the size (i.e. the diameter D) of the system, respectively, as reported in Fig. S3-b for $E_F = 0.4$ eV (β = 0.0031).[1]

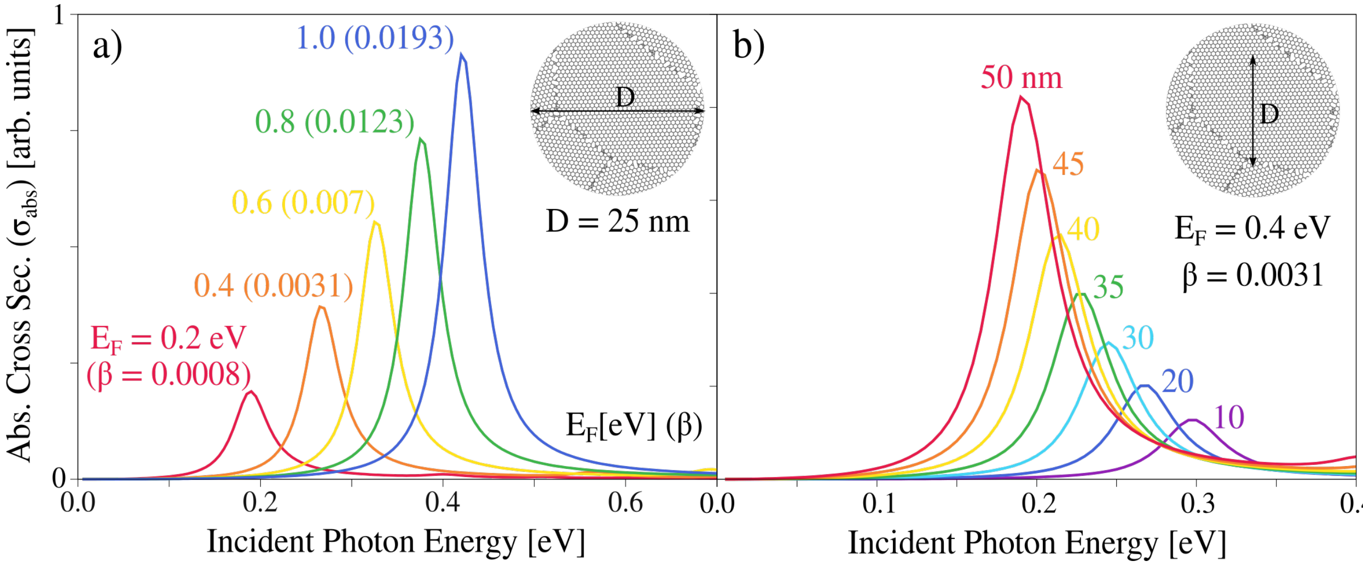

Figure S3: (**a**) ωFQ σ_{abs} for a graphene disk (D = 25 nm) as a function of E_F (from 0.2 to 1.0 eV; β from 0.0008 to 0.0193). (**b**) ωFQ σ_{abs} for a graphene disk with E_F= 0.4 eV (β= 0.0031) as a function of the disk's diameter (from 10 to 50 nm).

Also, by tuning both the size and the Fermi energy of the substrate by the same numerical factor, a plasmon degeneracy occurs (see Fig. S4), making the model capable of reproducing a peculiar property of graphene-based materials.[1,6] In other words, starting from an initial graphene disk with diameter D and Fermi level E_F, it is possible to model the same electron density over a disk with bigger/smaller diameter by increasing/decreasing its Fermi level by the same numerical factor. As example, in Fig. S4 the plasmon frequency degeneracy arising in two graphene disks with D = 25 nm ($E_F = 0.4$ eV, β = 0.0031) and $D = 50$ nm ($E_F = 0.8$ eV, $\beta = 0.0492$) is reported.

This feature can be exploited to describe the optical properties of very large nanostructures (in the scale of μm) by properly tuning the physical parameters of a smaller system. This is achieved by decreasing the electron density by means of the β parameter (see S5), in order to make PRF falling in the THz region.

In Fig. S5 the real and imaginary parts of σ_{2D} for a graphene sheet (L = 28 nm) are reported as a function

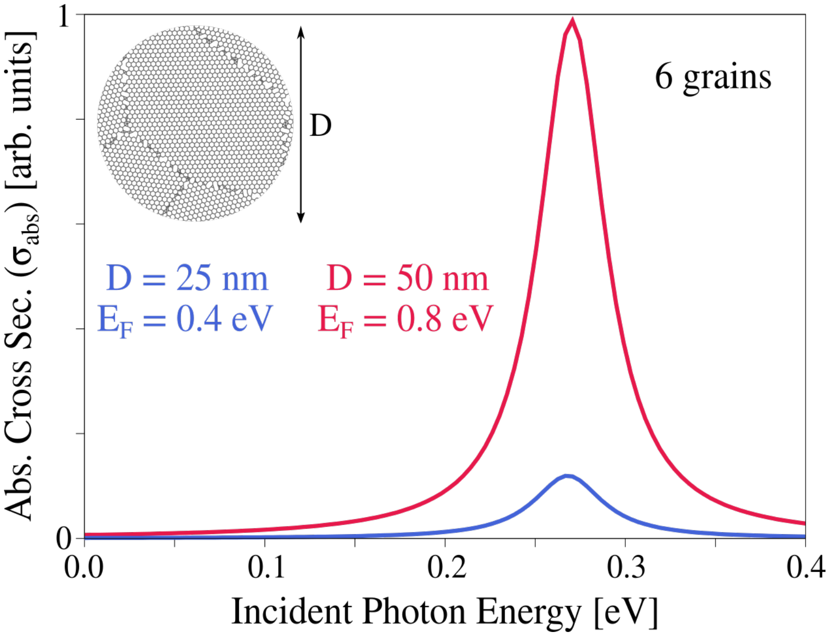

Figure S4: ωFQ σ_{abs} of two polycrystalline graphene disks with D = 25 nm and D = 50 nm, calculated by imposing $E_F = 0.4$ and $E_F = 0.8$ eV, respectively.

of the β parameter. By decreasing β (i.e. the electron density of the system and, in turn, the Fermi level) the PRF red shifts, resembling an increase in the system size. At a value of $\beta = 1.0 \cdot 10^{-14}$ the Drude limit, corresponding to the bulk conduction, is reached (see the black dotted line in Fig. S5).

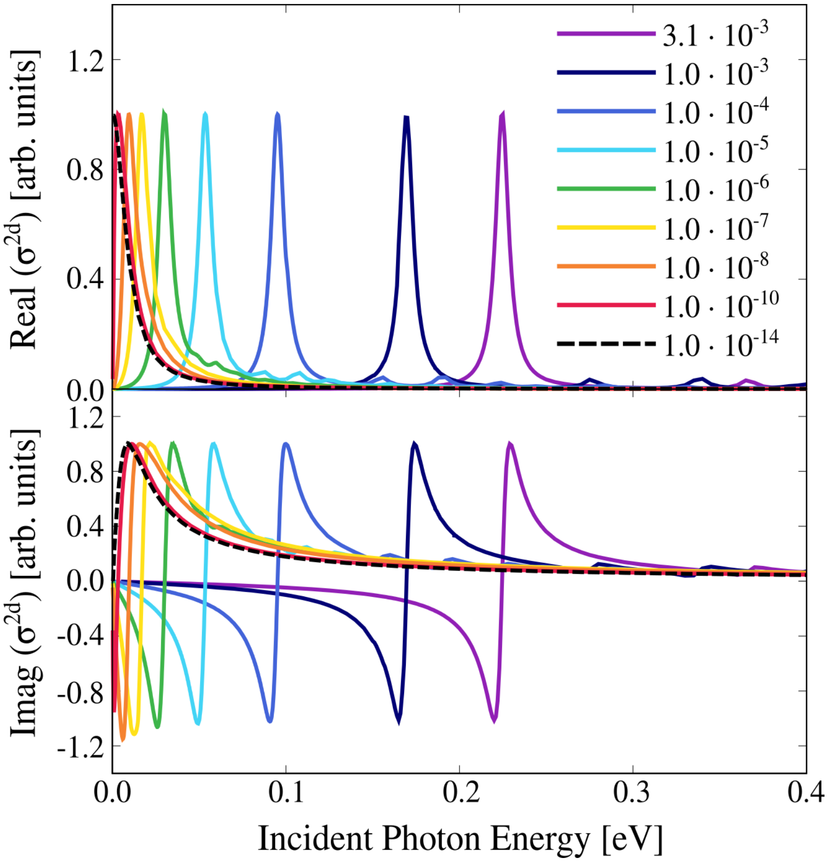

Figure S5: ωFQ real (top) and imaginary (bottom) conductivity (σ^{2d}) of a graphene square sheet (with side $L = 28$ nm, 30360 atoms) as a function of the β parameter.

S3.3 Fitting procedure

Once the real and imaginary parts of conductivity are calculated (see Eq. S4), the key parameters of the Drude-Smith model can be obtained by a fitting procedure. In particular, the fitting function is:

$$G = K \cdot G_0 E_F \frac{\tau_{DS}}{1 - i\omega\tau_{DS}} \left(1 - \frac{c}{1 - i\omega\tau_{DS}}\right) \tag{S7}$$

where G_0 is the quantum of conductance, E_F the Fermi level, c is the Drude-Smith constant,[7] τ_{DS} the transport scattering time (i.e. $\tau_{DS} = \tau/(1-c)$, where τ is the measured scattering time)[7] and K is an additional fitting parameter.

S3.4 Parametrization for the initial system

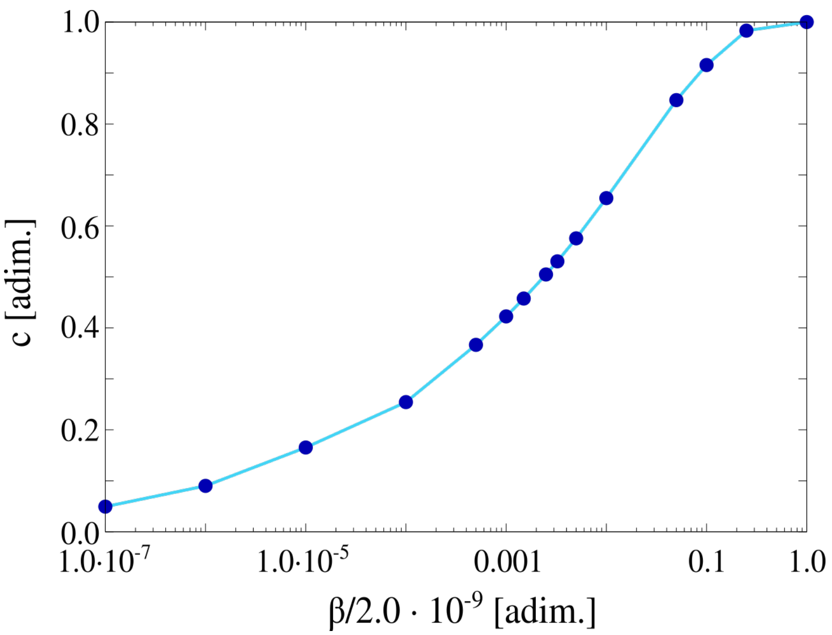

Figure S6: ωFQ Drude-Smith c parameter[7] as a function of the β parameter.

In Fig. S6, the dependence of the Drude-Smith parameter c as a function of the β parameter is graphically depicted. We select $\beta = 6.5 \cdot 10^{-12}$ in order to obtain an initial value of $c = 0.53$ (see Eq. S7) for the geometry at rest, as reported from the experimental counterpart (see Fig. S7).

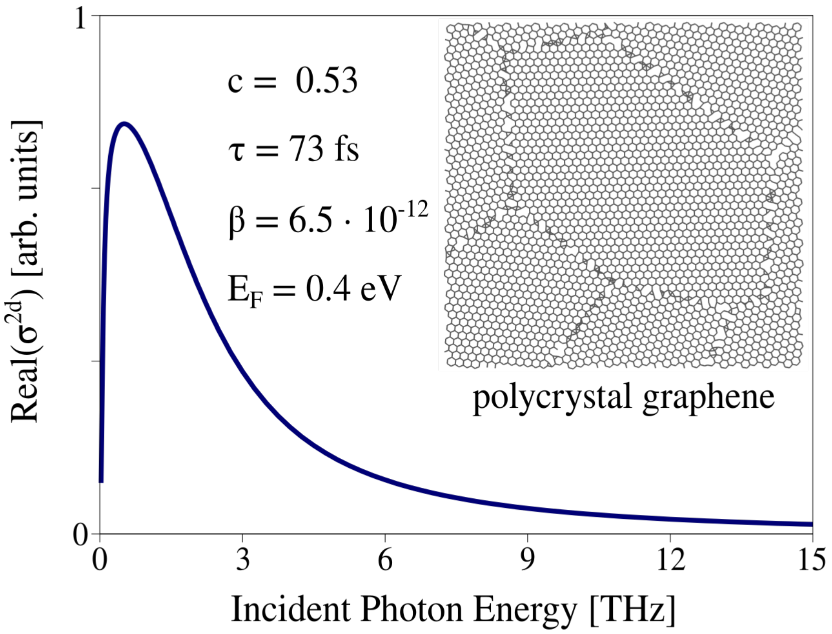

Figure S7: Real part of ωFQ conductivity for a multidomain graphene sheet. $\beta = 6.5 \cdot 10^{-12}$, $\tau = 73$ fs, E_F = 0.4 eV and c = 0.53.

S3.5 Computational Details

ωFQ[1] calculations were performed by using a stand-alone program, named nanoFQ written in Fortran95. The on-the-fly GMRES algorithm[8] was exploited for all systems considered in this work (root mean square error set to 1.0E-05). Polycrystalline graphene square sheets were obtained by using Atomsk.[9]

S3.6 Parameters of the Cocker approach[10] as a function of the applied strain

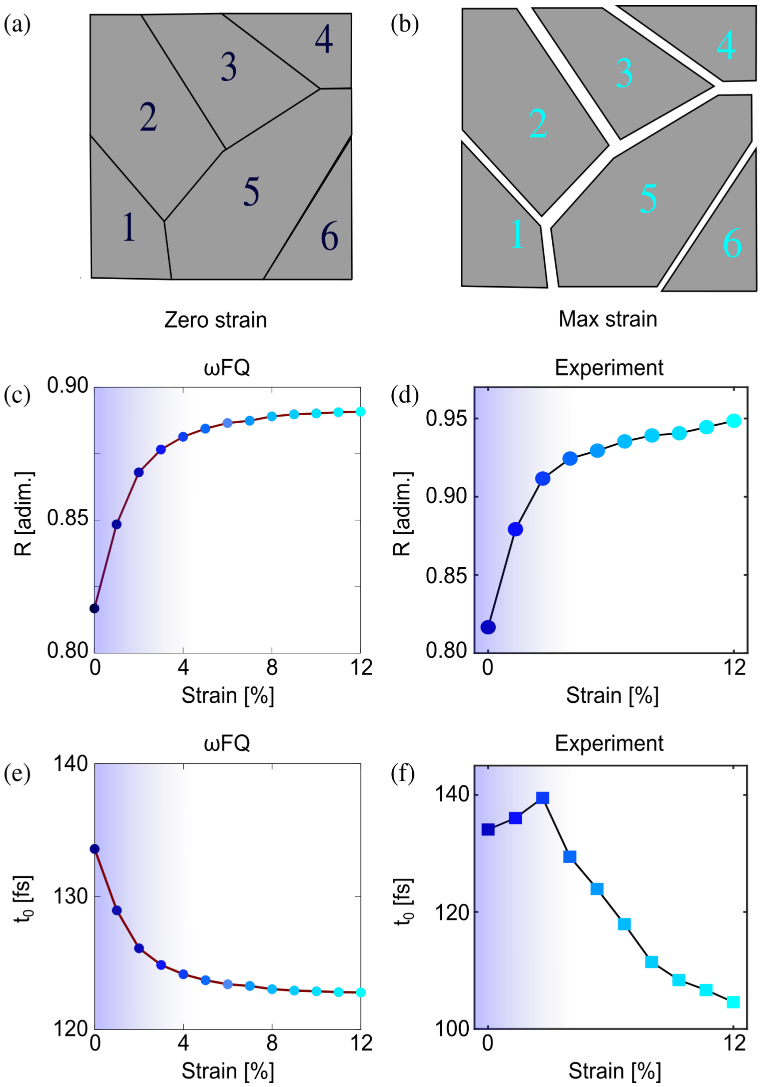

Figure S8: (a,b) Schematic depiction of a multidomain graphene sample at rest (a) and subjected to an isotropic strain (b). (c-f) Computed (c,e) and experimental (d,f) Cocker[10] parameters as a function of the applied strain.

S3.7 Role of the grain size as a function of the applied strain

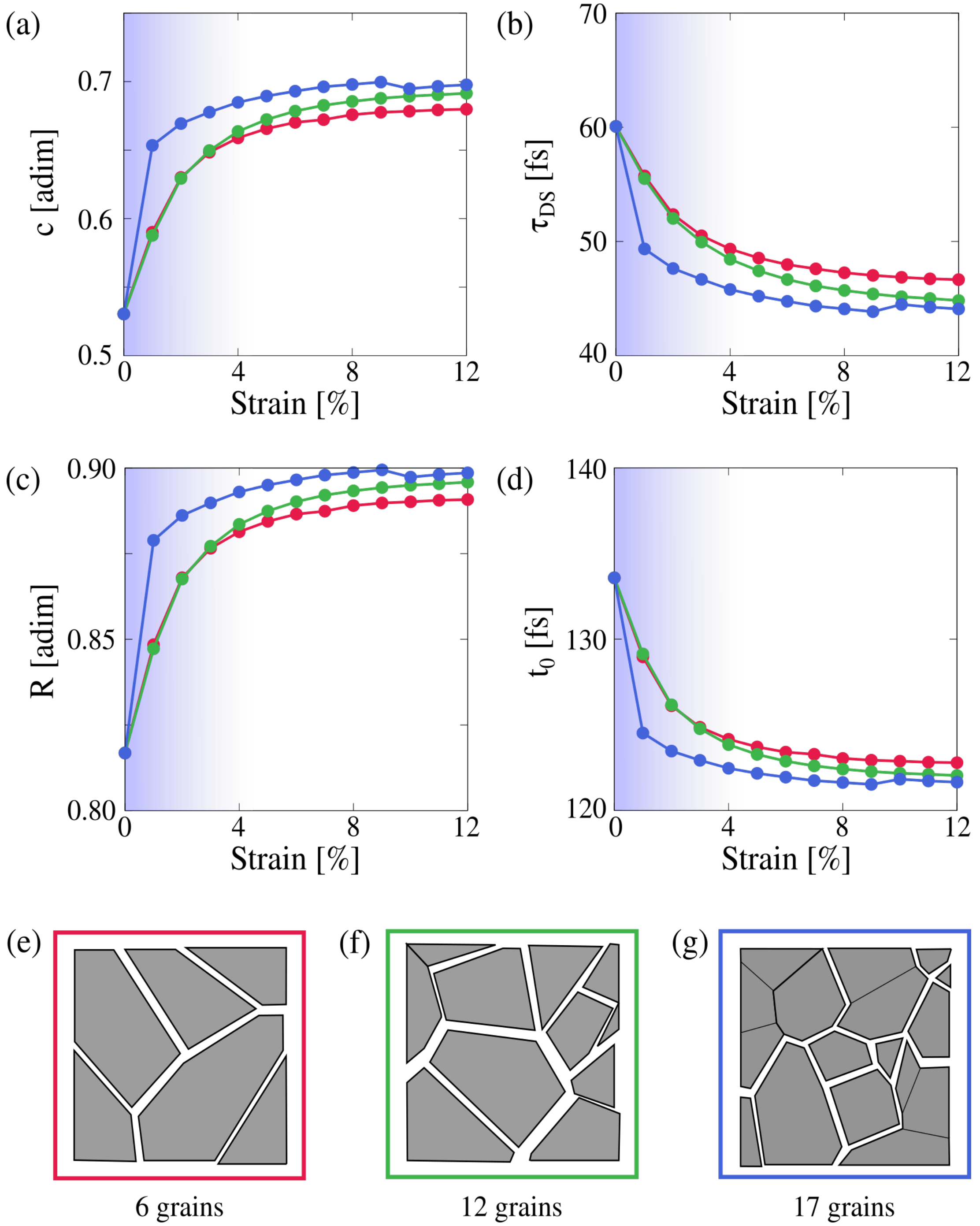

Figure S9: ωFQ computed values of c (a), τ_{DS} (b), R (c) and t_0 (d) parameters as a function of the applied strain for three different polycrystalline graphene sheets, constituted by 6 (e, red), 12 (f, green) and 17 (g, blue) grains.

S3.8 Alternative modeling of the applied strain

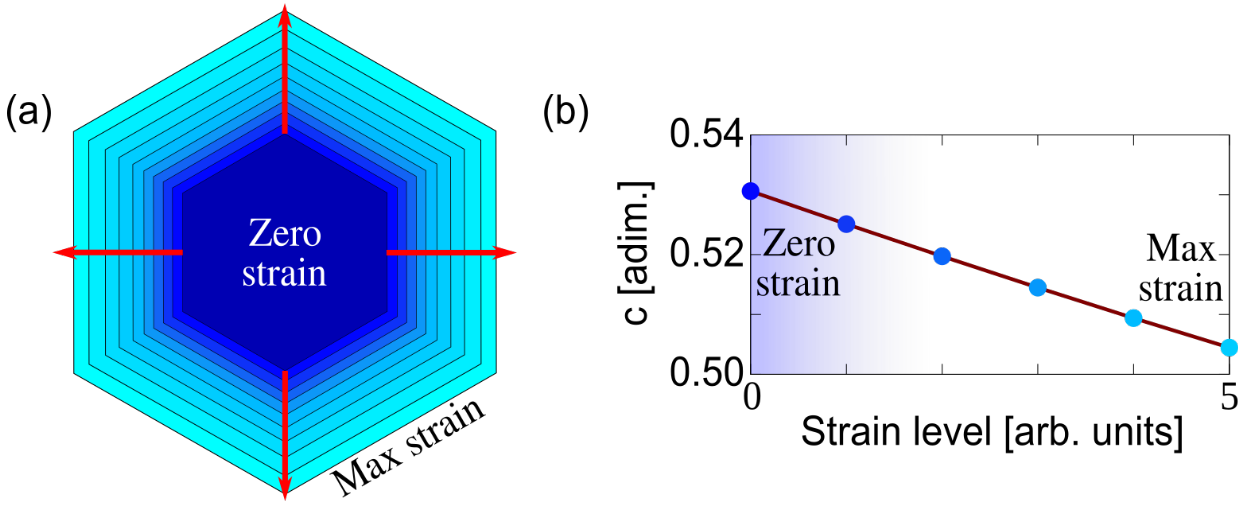

Figure S10: Alternative modeling of the applied strain as an elongation of the C-C bonds.(a) Different strain levels on a graphene ring. (b) ωFQ Drude-Smith c parameter as a function of the applied strain (from 1 to 5 %).

Table S1: C-C bond length for polycrystalline graphene as a function of the biaxial strain.

Strain [%]	C-C length [Å]
0	1.42
1	1.43
2	1.45
3	1.46
4	1.48
5	1.49

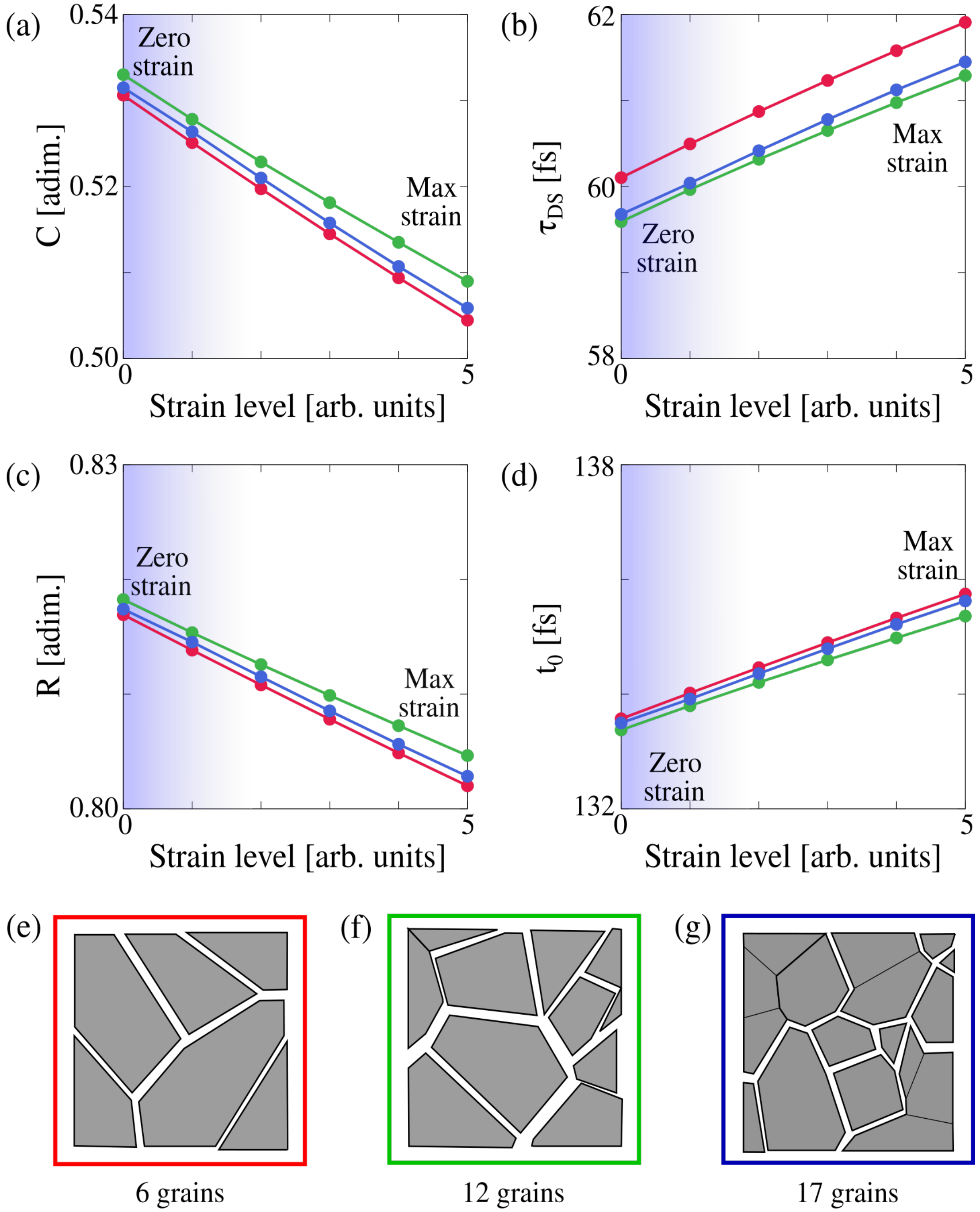

Figure S11: ωFQ computed values of c (a), τ_{DS} (b), R (c) and t_0 (d) parameters as a function of the strain level applied to a polycrystalline graphene sheet composed by 6 (e, red), 12 (f, green) and 17 (g, blue) grains respectively.

References

(1) Giovannini, T.; Bonatti, L.; Polini, M.; Cappelli, C. Graphene Plasmonics: Fully Atomistic Approach for Realistic Structures. *The journal of physical chemistry letters* **2020**, *11*, 7595–7602.

(2) Thongrattanasiri, S.; Manjavacas, A.; Garcia de Abajo, F. J. Quantum finite-size effects in graphene plasmons. *Acs Nano* **2012**, *6*, 1766–1775.

(3) Cox, J. D.; Silveiro, I.; García de Abajo, F. J. Quantum effects in the nonlinear response of graphene plasmons. *ACS nano* **2016**, *10*, 1995–2003.

(4) Fang, Z.; Thongrattanasiri, S.; Schlather, A.; Liu, Z.; Ma, L.; Wang, Y.; Ajayan, P. M.; Nordlander, P.; Halas, N. J.; García de Abajo, F. J. Gated tunability and hybridization of localized plasmons in nanostructured graphene. *ACS nano* **2013**, *7*, 2388–2395.

(5) Fang, Z.; Wang, Y.; Schlather, A. E.; Liu, Z.; Ajayan, P. M.; García de Abajo, F. J.; Nordlander, P.; Zhu, X.; Halas, N. J. Active tunable absorption enhancement with graphene nanodisk arrays. *Nano letters* **2014**, *14*, 299–304.

(6) Yu, R.; Cox, J. D.; Saavedra, J.; Garcia de Abajo, F. J. Analytical modeling of graphene plasmons. *ACS Photonics* **2017**, *4*, 3106–3114.

(7) Smith, N. Classical generalization of the Drude formula for the optical conductivity. *Physical Review B* **2001**, *64*, 155106.

(8) Lafiosca, P.; Giovannini, T.; Benzi, M.; Cappelli, C. Going beyond the limits of classical atomistic modeling of plasmonic nanostructures. *The Journal of Physical Chemistry C* **2021**, *125*, 23848–23863.

(9) Hirel, P. Atomsk: A tool for manipulating and converting atomic data files. *Computer Physics Communications* **2015**, *197*, 212–219.

(10) Cocker, T. L.; Baillie, D.; Buruma, M.; Titova, L. V.; Sydora, R. D.; Marsiglio, F.; Hegmann, F. A. Microscopic origin of the Drude-Smith model. *Physical Review B* **2017**, *96*, 205439.
